# Single-cell genetic expression of mutant GABA_A_ receptors causing Human genetic epilepsy alters dendritic spine and GABAergic bouton formation in a mutation-specific manner

**DOI:** 10.3389/fncel.2014.00317

**Published:** 2014-10-14

**Authors:** Pamela Lachance-Touchette, Mayukh Choudhury, Ana Stoica, Graziella Di Cristo, Patrick Cossette

**Affiliations:** ^1^Centre d'Excellence en Neuromique de l'Université de Montréal, Centre de Recherche, Centre Hospitalier de l'Université de Montréal, Université de MontréalMontréal, QC, Canada; ^2^Centre Hospitalier Universitaire Sainte-Justine, Université de MontréalMontréal, QC, Canada

**Keywords:** channelopathy, epilepsy, bouton formation, human genetics, GABA_A_ receptor, organotypic slice culture

## Abstract

Mutations in genes encoding for GABA_A_ receptor subunits is a well-established cause of genetic generalized epilepsy. GABA neurotransmission is implicated in several developmental processes including neurite outgrowth and synapse formation. Alteration in excitatory/inhibitory synaptic activities plays a critical role in epilepsy, thus here we investigated whether mutations in α1 subunit of GABA_A_ receptor may affect dendritic spine and GABAergic bouton formation. In particular, we examined the effects of three mutations of the *GABRA1* gene (D219N, A322D and K353delins18X) that were found in a cohort of French Canadian families with genetic generalized epilepsy. We used a novel single-cell genetic approach, by preparing cortical organotypic cultures from *GABRA1*^flox/flox^ mice and simultaneously inactivating endogenous *GABRA1* and transfecting mutant α1 subunits in single glutamatergic pyramidal cells and basket GABAergic interneurons by biolistic transfection. We found that *GABRA1*^−/−^ GABAergic cells showed reduced innervation field, which was rescued by co-expressing α1-A322D and α1-WT but not α1-D219N. We further found that the expression of the most severe *GABRA1* missense mutation (α1-A322D) induced a striking increase of spine density in pyramidal cells along with an increase in the number of mushroom-like spines. In addition, α1-A322D expression in GABAergic cells slightly increased perisomatic bouton density, whereas other mutations did not alter bouton formation. All together, these results suggest that the effects of different GABA_A_R mutations on GABAergic bouton and dendritic spine formation are specific to the mutation and cannot be always explained by a simple loss-of-function gene model. The use of single cell genetic manipulation in organotypic cultures may provide a better understanding of the specific and distinct neural circuit alterations caused by different GABA_A_ receptor subunit mutations and will help define the pathophysiology of genetic generalized epilepsy syndromes.

## Introduction

Genetic factors play a key role in the development and severity of genetic generalized epilepsy (GGE). Epilepsy-causing mutations have been identified in several GABA_A_ receptor (GABA_A_R) subunits, including α1, β3, γ2, and δ subunits (Baulac et al., 2001; Wallace et al., 2001; Cossette et al., 2002; Harkin et al., 2002; Kananura et al., 2002; Dibbens et al., 2004, 2009; Audenaert et al., 2006; Maljevic et al., 2006; Sun et al., 2008; Tanaka et al., 2008; Lachance-Touchette et al., 2010, 2011; Shi et al., 2010; Klassen et al., 2011; Carvill et al., 2013, 2014; Epi et al., 2013; Tian et al., 2013; Hancili et al., 2014; Ishii et al., 2014; Johnston et al., 2014). GABA_A_Rs are ligand-gated ion channels that are permeable to chloride and bicarbonate anions and mediate most of cortical inhibitory neurotransmission. Their molecular structure comprises of a heteropentameric protein complex assembled from 19 different subunits (α1–6, β1–3, γ1–3, δ, ε, π, θ, and ρ1–3). Although there is the potential for a high variability of combinations, the α1β 2γ2 is the most abundant and represents approximately 60% of all GABA_A_Rs in adult brain (Sieghart and Sperk, 2002). Mutations in the *GABRA1* gene are linked to a spectrum of endophenotypes of GGE syndromes as well as more severe forms of epilepsy associated with intellectual disability (Carvill et al., 2014). We previously reported D219N, A322D, K353delins18X mutations in families with autosomal dominant genetic generalized epilepsy (Cossette et al., 2002; Lachance-Touchette et al., 2011). Whether these mutations cause protein inactivation and thus loss of function is still unclear. Deletion of α1 in mice produced EEG spike-wave discharges and absence-like seizures (Arain et al., 2012). This mouse model recapitulates some of the epilepsy phenotypes that were reported in human carriers of *GABRA1* mutations (Cossette et al., 2002; Maljevic et al., 2006; Klassen et al., 2011; Lachance-Touchette et al., 2011). *In vitro* investigations in heterologous cells demonstrated that *GABRA1* mutants could affect mRNA transcript stability, cell surface GABA_A_R composition and channel gating properties (Cossette et al., 2002; Gallagher et al., 2004, 2007; Krampfl et al., 2005; Maljevic et al., 2006; Lachance-Touchette et al., 2011; Carvill et al., 2014). By expressing wild type or mutant α1 in heterologous cells, we previously showed that A322D and K353delins18X mutations reduced GABA-evoked currents amplitude by impairing α1β 2γ2 receptor surface expression due to endoplasmic reticulum retention (Krampfl et al., 2005; Lachance-Touchette et al., 2011). In addition, two *GABRA1* mutations (A322D, D219N) exhibited altered gating kinetic properties (Lachance-Touchette et al., 2011). Further, studies in cultured neurons revealed that α1-A322D mutation altered the kinetics and the amplitude of miniature inhibitory postsynaptic currents (mIPSCs) in pyramidal neurons (Ding et al., 2010). These data support the hypothesis that reduced inhibition underlies network hyperexcitability in GGE associated with GABA_A_R mutations.

On the other hand, GABA_A_R mutations may also alter specific developmental processes. Alterations in the number and strength of inhibitory and excitatory synapses are thought to contribute to epilepsy (Bernard, 2012). In addition, GABA transmission have been shown to play a key role during brain development, influencing virtually all developmental steps from neurogenesis to the establishment of neuronal connectivity (Gaiarsa and Porcher, 2013; Kilb et al., 2013). Focusing in particular on synaptogenesis, recent studies demonstrated in organotypic cortical slices that endogenous GABA regulates axonal branching and synapse formation of cortical basket cells—a prominent class of GABAergic neurons—through the activation of GABA_A_ and GABA_B_ receptors (Chattopadhyaya et al., 2007; Baho and Di Cristo, 2012; Wu et al., 2012). GABAergic transmission can also play a critical role in excitatory synapse development. In pyramidal neurons of the cerebral cortices, excitatory synaptic inputs are made on small dendritic protrusions, called dendritic spines. Hayama et al. (2013) showed that dendritic spine shrinkage and elimination can be promoted either by uncaging of a caged GABA compound that mimics IPSCs or by tonic application of a GABA_A_ agonist, muscimol. Whether and how *GABRA1* mutations affect dendritic spines and GABAergic bouton formation, thus contributing to the epilepsy phenotype has not been so far examined.

So far, the vast majority of mutations in GABA_A_R subunits causing Human epilepsy are associated with loss-of-function, when assessing gating properties of the GABA-evoked currents *in vitro* (Macdonald and Kang, 2009). However, review of functional studies on GABRA mutations in heterologous cell system revealed controversial findings between different groups (reviewed in Cossette et al., 2012). For example, for long time no consensus was reached regarding the impact of *GABRG2* missense mutations on GABA currents amplitude or kinetics as well as cell surface expression by using heterologous cell culture. Only the generation of a mouse model harboring the γ2 point mutation (R82Q) dissipated all these ambiguities (Tan et al., 2007). The emergence of massive gene-sequencing studies will generate an enormous amount of data, on the other hand developing mouse knock-in models for each new GABRA mutations is unrealistic, both because it is time consuming and far too expansive.

Here, we propose of using single cell genetic manipulation to investigate the effects of different mutations of GABA_A_ α1 subunit on both dendritic spine and GABAergic bouton formation in cortical organotypic slices, which maintain the three dimensional structure of the brain tissue and the tight spatial relationships between different cell types. In particular, we analyzed the density and morphology of pyramidal cell dendritic spines, which are the preferential postsynaptic site of glutamatergic synapses. We also examined the axon morphology and bouton density of basket cells, which are the most prominent type of GABAergic interneurons in the cortex.

## Materials and methods

### Mice

Funder mice B6.129(FVB)-*Gabra1*^tm1Geh^/J, first described in Vicini et al. (2001), were kindly gifted by Dr. Rudolph (McLean Hospital, Harvard Medical School) (Vicini et al., 2001). They were bred to establish a colony in the animal facility at the *Centre de recherche du Centre hospitalier de l'Université de Montréal* (CRCHUM). All mice were housed under standard pathogen-free conditions in a 12 h light/dark cycle with *ad libitum* access to sterilized laboratory chow diet. Animals were treated in accordance with Canadian Council for Animal Care and protocols were approved by the Animal Care Committee of the CRCHUM and of CHU Ste-Justine Research Center. B6.129(FVB)-*Gabra1*^tm1Geh^/J mice were previously produced in a mixed background. The background was characterized with a microsatellite panel consisting of 110 markers spread across the genome at about 15 cM intervals and was confirmed to be 99.08% congenic to C57BL/6J background (Charles River, NY). B6.129(FVB)-*Gabra1*^tm1Geh^/J mice possess three *loxP* sites on both sides of the α1 exon encoding an essential transmembrane domain of GABA_A_ receptor.

### DNA constructs

P_*G*67_-GFP was generated by subcloning of a 10 kb region of *Gad1* gene promoter by gap repair in front of the GFP coding region in pEGFP (Clontech) as previously described (Chattopadhyaya et al., 2004). We subcloned CRE, *GABRA1*-A322D, *GABRA1*-D219N, *GABRA1*-K353delins18X constructs (Cossette et al., 2002; Lachance-Touchette et al., 2011) in P_*G*67_ vector by using restriction site Pme1 via sequence and ligation–independent cloning method (SLIC) (Li and Elledge, 2007). All constructs were sequenced to confirm the presence of the mutations and to exclude any other variants that may have been introduced during PCR amplification.

### Slice culture and biolistic transfection

Slice culture preparation was done as described by Stoppini et al. (1991). Postnatal day 4 or 5 (P4 or P5) mouse pups were decapitated, and brains were rapidly removed and immersed in ice-cold culture medium (containing DMEM, 20% horse serum, 1 mM glutamine, 13 mM glucose, 1 mM CaCl_2_, 2 mM MgSO_4_, 0.5 μm/ml insulin, 30 mM HEPES, 5 mM NaHCO_3_, and 0.001% ascorbic acid). Coronal brain slices of the occipital cortex, 400 μm thick, were cut with a Chopper (Stoelting, Wood Dale, IL). Slices were then placed on transparent Millicell membrane inserts (Millipore, Bedford, MA), usually three slices/insert, in 30 mm Petri dishes containing 0.75 ml of culture medium. Finally, the slices were incubated in a humidified incubator at 34°C with a 5% CO_2_-enriched atmosphere and the medium was changed three times per week. All procedures were performed under sterile conditions. Constructs to be transfected were incorporated into “bullets” that were made using 1.6 μm gold particles coated with a total of ~60 μg of the DNA(s) of interest. These bullets were used to biolistically transfect slices by Gene gun (Bio-Rad, Hercules, CA) at high pressure (180 ψ), and the transfected slices were incubated for 8 days *in vitro* under the same conditions as described above, before imaging. For each experimental group, cortical slices were prepared from at least three mice. On average 6–7 neurons were transfected per cortical organotypic slice. The majority of neurons labeled by this promoter were parvalbumin-positive basket cells (as described in Chattopadhyaya et al., 2004, 2007), while a minority (~10%) were pyramidal cells. Pyramidal cells were recognized by the complexity of their dendritic arbor, including an apical dendrite, and the presence of numerous dendritic spines.

### Imaging and spine analysis of pyramidal cells

Pyramidal neurons were imaged using a Leica confocal microscope SPE (63X glycerol immersion objective; NA 1.3). At least 6 labeled pyramidal neurons, characterized by the presence of a well defined apical dendrite, were randomly selected from cortical layers 2/3 and 5. Image stacks of basal dendrites were acquired with a z-step of 0.5 μm and then reconstructed in 3D with Neurolucida (MicroBrightField) software. Cortical pyramidal cells from at least four animals were used for each experimental condition. Dendritic length, total spine density, spine morphology and spine length were quantified using NeuroExplorer software (MicroBrightField). Mushroom spines were defined as spine with a neck and bearing a head, which was at least twice as large as the neck. Thin spines were defined as dendritic protrusions shorter than 5 μm and lacking a clearly defined head. All quantifications were done blind to the treatment.

### Analysis of basket cell innervation

We quantified two aspects of basket cell axon innervation field (1) the extent of perisomatic innervation around single neuronal somata (terminal branching and perisomatic GFP-positive bouton density) and (2) the percentage of potentially innervated cells in the field (percentage of innervation). We have previously shown that the vast majority of GFP-labeled boutons in our experimental condition most likely represent presynaptic terminals, by localization of pre- and post-synaptic markers and electron microscopy (Chattopadhyaya et al., 2004, 2007; Wu et al., 2012). For each experimental group, we took care to acquire an equal number of basket cells localized in layers 2/3 and 5/6 of the cortex. We acquired at least two confocal stacks of each basket cell axon arbor in the first 150 μm from the basket cell soma using a 63X glycerol objective (NA 1.3, Leica) and a Leica TCS SPE confocal microscope. The typical confocal stack size was 116.4 × 116.4 μm with an average depth of 40–70 μm and a z-step of 1 μm. Analysis of basket cell perisomatic innervation and bouton size was performed essentially as described by Chattopadhyaya et al. (2013). Briefly, in our Three-Dimensional Sholl analysis, Sholl spheres with a 1 μm increment from the center of a pyramidal soma were used to quantify basket axon terminal branch complexity and bouton density around the pyramidal cell soma. Axon branch complexity around a single pyramidal cell soma was quantified by the average number of intersections between basket cell axons and the Sholl spheres in the first 9 μm from the center of the pyramidal cell soma. We choose 9 μm as the limiting radius for a Sholl sphere because it approximates the average pyramidal cell soma diameter measured from pyramidal neurons immunostained with NeuN antibody. Between 10 and 15 pyramidal neurons were analyzed for each basket cell. Bouton density around each pyramidal cell soma was measured within the same set of Sholl spheres and averaged among pyramidal neurons for each condition. Only pyramidal cell somata with Sholl spheres, which intersected the basket cell axon in the first 9 μm from the center of their soma, were taken for analysis. Using this approach, we obtained an unbiased estimate of the number of presumptive boutons on individual labeled pyramidal cell soma. The percentage of neuron somata innervated by a basket cell was defined in a confocal stack by the number of NeuN-positive cells contacted by at least one GFP-positive-bouton divided by the total number of NeuN-positive cells. This was repeated over all the fields of each basket axon and the results were averaged (Chattopadhyaya et al., 2013).

All data were first averaged per basket cell, statistical analysis was then done using the number of basket cells as *n*.

### Statistical analysis

Differences between groups were assessed with One-Way ANOVA followed by *post-hoc* Holm–Sidak test for normally distributed data or One-Way ANOVA followed by *post-hoc* Kruskal–Wallis test for not-normally distributed data. The cells analyzed derived from at least three different sets of experiments. Data are expressed in term of mean ± standard error of mean (SEM).

## Results

*GABRA1* is broadly expressed in the nervous system and GABA_A_R-mediated signaling plays multiple roles during development (Rossignol, 2011). In order to examine how different *GABRA1* mutants may affects the formation of dendritic spine and GABAergic bouton formation, we used a transgenic mouse carrying a conditional allele of *GABRA1* (Vicini et al., 2001), which allows cell-type and developmental-stage restricted knockdown of *GABRA1* synthesis. In this floxed-*GABRA1* mouse (*GABRA1^flox/flox^*), Cre-mediated recombination results in excision of exon 8, causing a shift in reading frame and premature termination of translation.

**Figure 1:**
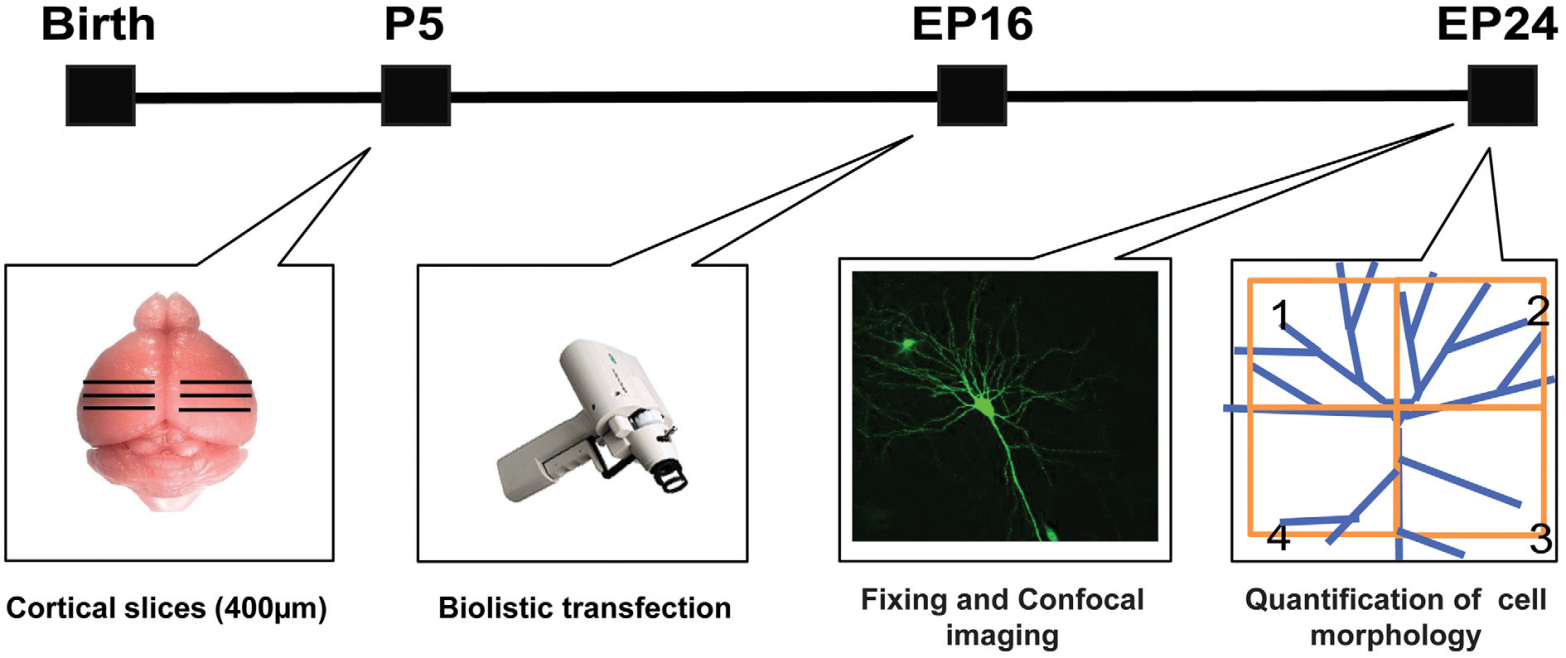
**Schematic of the experimental approach**.

**Figure 2:**
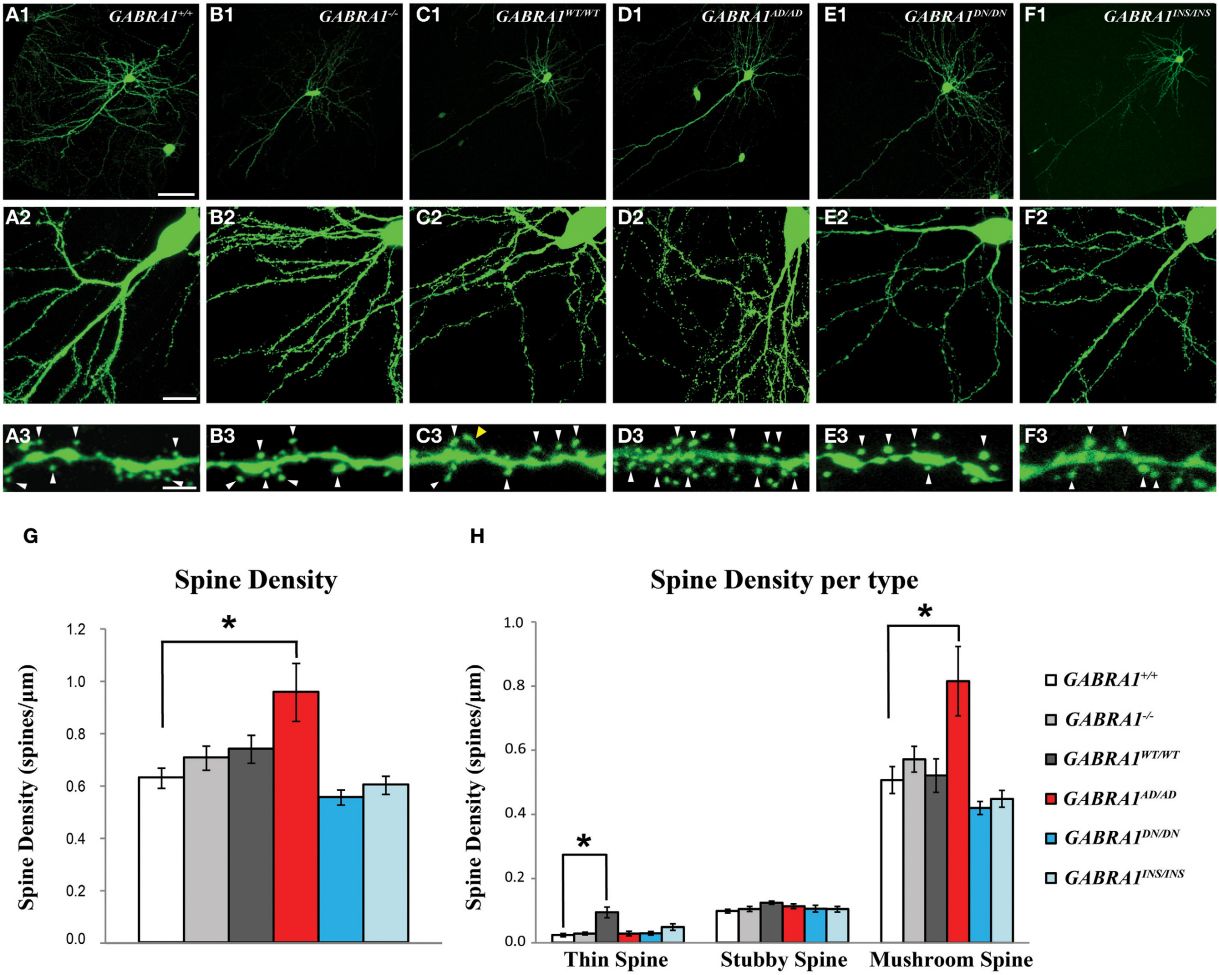
**α1-A322D expression induces a significant increase in the number and maturation of dendritic spines in cortical pyramidal cells. (A1–A3)** Confocal images showing pyramidal cells transfected with GFP (green) alone (control, *GABRA1^+/+^* cells) or **(B1–B3)** GFP and CRE (*GABRA1*^−/−^) to knockdown endogenous α1 subunits, or GFP-CRE and either one of the wild-type or mutants α1 **(C1–F3)** to investigate the effects of different α1 mutants on spine density and morphology, in organotypic cultures. **(A3–F3)** High-magnification images of dendrites from pyramidal cells in **A2–F2**. White arrowheads indicates mushroom spines, yellow arrowhead indicate a thin spine. **(G,H)** α1-A322D mutant pyramidal cells show significantly increased density of total **(G)** and mushroom-like spines **(H)** compared to control age-matched pyramidal cells (One-Way ANOVA; ^*^p < 0.05). α1-WT expression induces a significant increase in thin-like spines (**H**, One-Way ANOVA; ^*^p < 0.05). GFP *n* = 7; GFP-CRE *n* = 9; GFP-CRE-WT *n* = 7; GFP-CRE-A322D *n* = 7; GFP-CRE-D219N *n* = 6; GFP-CRE-K353delins18X *n* = 7 pyramidal cells. Scale bars: **A1–F1**, 50 μm; **A2–F2**, 10 μm; **A3–F3**, 5 μm.

**Figure 3:**
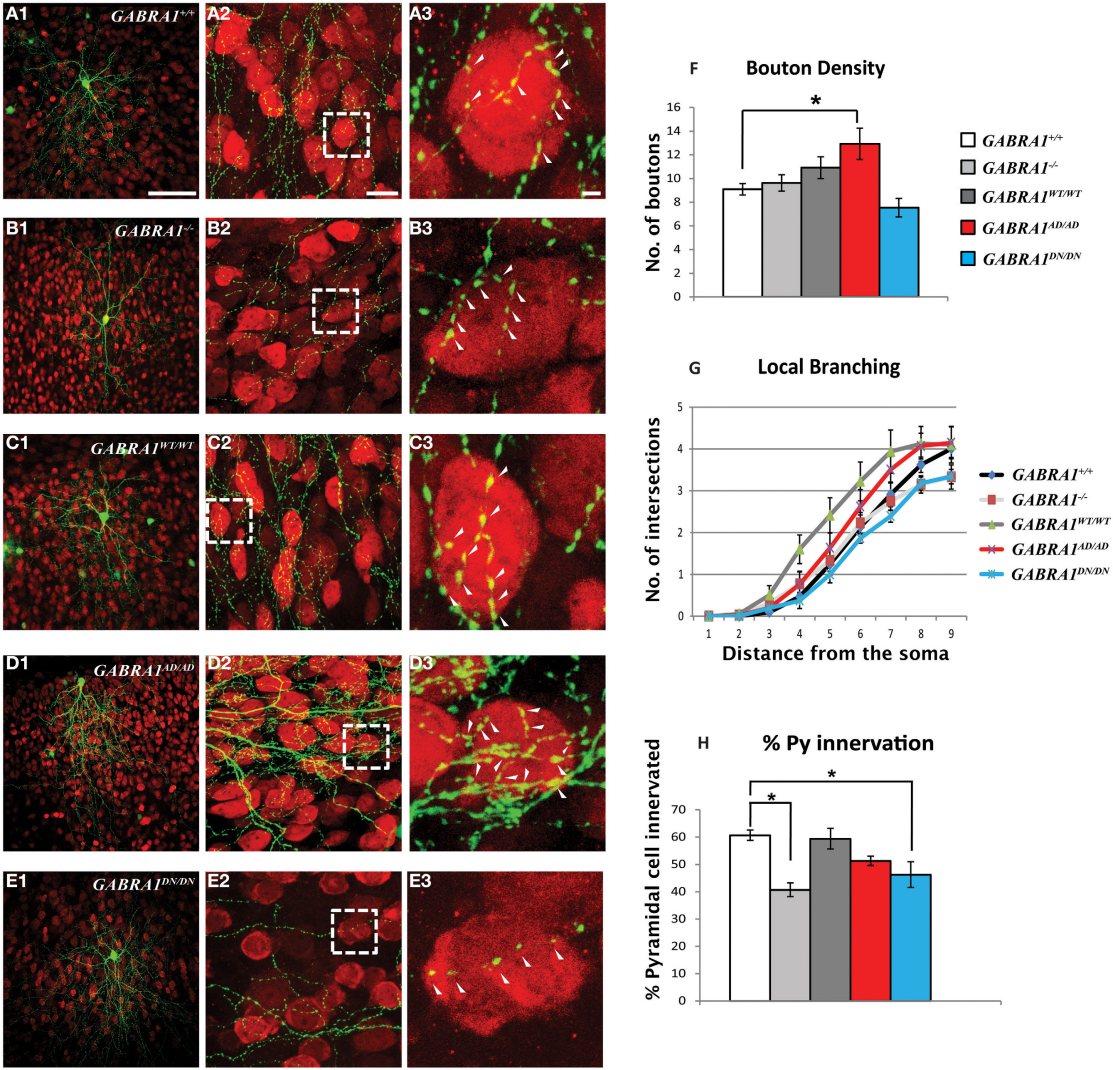
**α1-A322D expression induces a significant increase in boutons formed by GABAergic cortical basket cells. (A–E)** Low **(A1–E1)** and high magnification **(A2–E2)** confocal images showing basket cells transfected with GFP (green) alone (**A***, GABRA1^+/+^*cells) or GFP and CRE (**B***, GABRA1*^−/−^ cells), or GFP-CRE and either one of the wild-type or mutants α1 subunits **(C–E)**. Basket cells form terminal axon branching bearing numerous presynaptic boutons around NeuN (red)-positive somata (arrowheads). **A3–E3** are high-magnification images from boxed areas in **A2–E2**. **(F)** *GABRA1*^−/−^ basket cells transfected with α1-A322D show significant increase in bouton density **(F)**. Local branching **(G)** does not differ across the groups. **(H)** *GABRA1^−/−^* basket cells contact less pyramidal somata compared to age-matched basket cells. This deficit is rescued by the expressions of α1-wild-type or α1-A322D but not of α1-D219N (One-Way ANOVA; ^*^p < 0.05). GFP *n* = 6; GFP-CRE, *n* = 6; GFP-CRE-WT, *n* = 6; GFP-CRE-A322D, *n* = 6; GFP-CRE-D219N, *n* = 4 basket cells. Scale bars: **A1–E1**, 50 μm; **A2–E2**, 10 μm; **A3–E3**, 5 μm.

To inhibit *GABRA1* expression in isolated pyramidal neurons and GABAergic basket cells and simultaneously label their dendritic and axonal arbors at high resolution, we used a previously characterized promoter region P_G67_ (Chattopadhyaya et al., 2004) to express either Cre recombinase together with GFP (P_G67_-GFP/Cre) or GFP alone (P_G67_-GFP) in cortical organotypic cultures of *GABRA1^flox/flox^* mice (Figure 1). For pyramidal neurons, we focussed our analysis on dendritic spines, because dendritic spine alterations have been observed both in experimental animal models of epilepsy (Wong, 2005; Ma et al., 2013) and in human epilepsy patients (Multani et al., 1994; Isokawa, 2000). GABAergic basket cells (BCs), which represent about 40% of all cortical GABAergic cells in rodents, form synapses onto the somata and proximal dendrites of excitatory pyramidal cells. Because of the perisomatic localization and strength of their synapses, BCs strongly control the firing output of pyramidal cells and are thought to be important contributors to the maintenance of the overall excitation/inhibition balance in the cortex (Haider and McCormick, 2009). Further, BCs could act as a gate to prevent runaway excitation, which underlies the propagation of epileptiform activity (Trevelyan et al., 2007). Previous studies have shown that the basic features of dendritic spine formation and of the maturation of perisomatic innervation by BCs onto pyramidal cells are retained in cortical organotypic cultures (Dunaevsky et al., 1999; Chattopadhyaya et al., 2004; Di Cristo et al., 2004). We genetically manipulated pyramidal cells and BCs between the third and fourth postnatal week during which a significant and stereotyped maturation of BCs perisomatic innervation occurs (Chattopadhyaya et al., 2004, 2007; Di Cristo et al., 2007). Pyramidal cells from *GABRA1^flox/flox^* cultures transfected with P_G67_-GFP/Cre (referred here on as *GABRA1*^−/−^ cells) from equivalent postnatal day 16 (EP16, P4 + 12 days *in vitro*) to EP24 showed no significant alterations in the overall spine density and morphology compared to age-matched control transfected only with P_G67_-GFP (Figures 2G–H; Supplementary Figure 1; total spine density *GABRA1^+/+^* vs. *GABRA1*^−/−^; 0.63 ± 0.04 vs. 0.71 ± 0.05 spine/μm; *p* > 0.05). *GABRA1*^−/−^ BCs showed a significant reduction in the number of contacted target cells (Figure 3H; *GABRA1^+/+^* vs. *GABRA1*^−/−^; 61 ± 2% vs. 41 ± 3%; *p* < 0.05). In turn, the perisomatic innervations they formed around contacted neurons did not differ from those formed by control age-matched BCs, in term of bouton density or terminal branching (Figure 3F; *GABRA1^+/+^* vs. *GABRA1*^−/−^; 9.1 ± 0.5 vs. 9.6 ± 0.7 boutons/soma; *p* > 0.05). The axon density and average internode axon length were also not significantly different between these two groups (Supplementary Figure 2), thus suggesting that knockdown of *GABRA1* in this developmental time window did not affect overall axon growth.

To explore whether and how specific *GABRA1* mutants associated with GGE affect pyramidal cell spine and BCs GABAergic bouton formation, we inactivated the endogenous *GABRA1* alleles and simultaneously re-introduced either *GABRA1^WT/WT^*or each of the *GABRA1* mutant separately in single pyramidal and BCs from EP16–24. We choose this approach because global *GABRA1* manipulations may alter the excitation/inhibition balance of the whole slice, therefore making it difficult to distinguish between specific effects of distinct *GABRA1* mutants and unspecific, generalized effects of altered neuronal activity. In our experimental model, *GABRA1* was deleted only in sparse neurons in an otherwise wild-type background. It is therefore unlikely that the overall excitation levels in the slices was altered. A second critical advantage of our single-cell labeling/genetic manipulation approach is that we could visualize, reconstruct and quantify at high-resolution the dendritic and axonal arbors of single neurons with their putative boutons.

*GABRA1^WT/WT^* expression in *GABRA1*^−/−^ pyramidal cells did not alter overall spine density (Figure 2G; *GABRA1^+/+^* vs. *GABRA1^WT/WT^*; 0.63 ± 0.04 vs. 0.74 ± 0.05 spine/μm *p* > 0.05), although we observed a slight increase in the density of thin spines (Figure 2H; *GABRA1^+/+^* vs. *GABRA1^WT/WT^*; 0.02 ± 0.005 vs. 0.09 ± 0.02 thin spines/μm; *p* < 0.001). Importantly, *GABRA1^WT/WT^* expression rescued the number of target cells contacted by each *GABRA1*^−/−^ BC (Figure 3H*; GABRA1^+/+^* vs. *GABRA1^WT/WT^*; 61 ± 2% vs. 59 ± 4%; *p* > 0.05 and Figure 3H; *GABRA1*^−/−^ vs. *GABRA1^WT/WT^*; *41* ± 3% vs. 59 ± 4%; *p* < 0.001) suggesting that biolistic transfection of *GABRA1^WT/WT^* under the P_G67_ promoter can drive the expression of enough protein to rescue *GABRA1* deficits in single cells.

Interestingly, we found that α1-A322D expression in *GABRA*^−/−^ pyramidal cells specifically and significantly increased both total spine density (Figure 2G; *GABRA1^+/+^* vs. *GABRA1^AD/AD^*; 0.63 ± 0.04 vs. 0.9 ± 0.1 spines/μm; *p* < 0.05) and the proportion of mushroom-like spines on pyramidal cells basal dendrites (Figure 2H; Supplementary Figure 1; *GABRA1^+/+^* vs. *GABRA1^AD/AD^*; 0.48 ± 0.05 vs. 0.8 ± 0.1 mushroom spines/μm; *p* < 0.05). As dendritic spines are the preferential site for glutamatergic synapse formation and mushroom spines in particular tend to show larger EPSCs compared to other spine types (Lee et al., 2012), these data suggest that α1-A322D expression may increase both the number and strength of excitatory synapses. In parallel, we found that α1-A322D expression in *GABRA1*^−/−^ BCs rescued the loss of innervated targets caused by *GABRA1* deletion (Figure 3H; *GABRA1^+/+^* vs. *GABRA1^AD/AD^*; 61 ± 2% vs. 51 ± 2%; *p* > 0.05) and further increased the number of GABAergic boutons formed by BCs onto target cell somata compared to age-matched controls (Figure 3F; *GABRA1^+/+^* vs. *GABRA1^AD/AD^*; 9.1 ± 0.5 vs. 13 ±1 boutons/soma; *p* < 0.05), suggesting that α1-A322D expression can increase the formation of GABAergic boutons.

Finally, we found that the expression of the other mutants, α1-D219N or α1-K353delins18X, had no effects on spine density and morphology in *GABRA1*^−/−^ pyramidal cells. On the other hand, α1-D219N expression failed to rescue the loss of innervated targeted cells caused by *GABRA1* deletion (Figure 3H; *GABRA1^+/+^* vs. *GABRA1^DN/DN^*; 61 ± 2 vs. 46 ± 5%; *p* < 0.001) and showed a trend toward reduced bouton density, which however did not reach significance (Figure 3F; *GABRA1^+/+^* vs. *GABRA1^DN/DN^*; 9.1 ± 0.5 vs. 7.5 ± 0.8 boutons/soma; *p* > 0.05). In summary, our data show a remarkably *GABRA1* mutant-specific effects on both dendritic spine and GABAergic bouton formation.

## Discussion

All together, our data show for the first time that different *GABRA1* mutations associated with familial autosomal dominant GGE can affect dendritic spine and GABAergic bouton formation in a mutation-specific manner. Interestingly, *GABRA1* deletion in single pyramidal neurons did not affect their dendritic spine density or morphology, likely due to the compensatory action of other α1 subunits of GABA_A_R. Consistently, α2 and α3 proteins were expressed at higher-level in the cerebral cortex of *GABRA1*-KO mice (Bosman et al., 2005; Zeller et al., 2008). In the same mouse model, global deletion of the α1 subunit triggered an increase in the density of postsynaptic sites expressing α3 subunit in the molecular layer of the cerebellum, which has been interpreted as a reorganization of cerebellar networks (Kralic et al., 2006). On the other hand, *GABRA1* deletion reduced the extent of BC innervation field in a cell-autonomous fashion (Figure 3), indicating that compensatory expression of other alpha subunits may not occur in GABAergic cells or that changes in inhibitory inputs caused by the presence of GABA_A_R lacking the α1 subunit may alter BC development. In fact, it has been shown that the maturation of the innervation field of GABAergic BCs is exquisitely dependent on neuron excitability and GABA release (Chattopadhyaya et al., 2007; Baho and Di Cristo, 2012; Wu et al., 2012). Consistent with this hypothesis, Purkinje cells from *GABRA1*^−/−^ mice lacked spontaneous and evoked IPSCs (Fritschy and Panzanelli, 2006). In addition, stellate cell synapses on Purkinje cells dendrites were reduced by 75% in the same mouse model (Fritschy and Panzanelli, 2006). Unexpectedly, the expression of α1-WT in a knock-out background (*GABRA1*^−/−^ pyramidal neurons) increased the formation of thin spines, which are generally thought to represent immature/new synapses. One possibility is that the overexpression of α1-WT causes excess inhibition, which in turn promotes the formation of new excitatory synapses (Queenan et al., 2012). Quantitative analysis of inhibitory and excitatory inputs onto transfected neurons will be necessary to clarify this issue and will be the focus on future studies.

Surprisingly, we found that different α1 mutants have very different impacts on the development of GABAergic boutons and dendritic spines. The *GABRA1* mutant that showed the most dramatic effects on both pyramidal cell spines and basket cell innervation field was α1-A322D. This observation is consistent with previous electrophysiological studies showing that this mutation has more severe effect *in vitro* on the gating properties of the GABA-evoked currents, compared to other *GABRA1* missense mutations (Macdonald et al., 2010; Lachance-Touchette et al., 2011). One possibility is that α1-A322D may act as dominant negative. Using cell cultures, Ding et al. (2010) showed that α1-A322D reduced the overall surface expression of GABA_A_R by associating with the wild type subunits within the endoplasmic reticulum and preventing them from trafficking to the cell surface (Ding et al., 2010; Lachance-Touchette et al., 2011). Reduction in cell surface expression of GABA_A_R resulted in decreased postsynaptic inhibition (Ding et al., 2010), which may in turn facilitate long-term potentiation (LTP) of excitatory synapses (Carlson et al., 2002). One of the main effects of LTP is the increase in AMPA-receptor density at postsynaptic sites on dendritic spines (Liu et al., 2005; Whissell et al., 2013), which correlate with the presence of more mature mushroom spines characterized by large heads (Luscher et al., 2000), consistently to what we observed (Figure 2). Similarly, reduction of inhibition onto BCs could promote GABA release and, subsequently, formation of GABAergic boutons (Chattopadhyaya et al., 2007; Baho and Di Cristo, 2012). Therefore, these results suggest that altered excitatory/inhibitory synaptic balance may be partially responsible for the increased excitability of cortical networks in human carriers of α1-A322D.

Interestingly, α1-D219N expression in *GABRA1*^−/−^ BC was unable to rescue the deficits in their innervation field, while it did not affect dendritic spine density. Our prior works showed that GABA_A_Rs containing α1-D219N were trafficked to the membrane and that mutation altered GABA_A_ receptor gating kinetics. In particular, GABA_A_Rs containing α1-D219N have slower desensitization rates and faster off-kinetics compared to wild-type receptors (Lachance-Touchette et al., 2011). It is therefore possible that reduced inhibition may be partially responsible for both altered development of GABAergic cells and increased excitability of neuronal circuits in human carriers of α1-D219N. Finally, the expression of α1-K353delins18X did not affect any of the developmental events we analyzed. We have previously reported that this frameshift mutation altered the downstream amino acid sequence and resulted in the introduction of a premature translation–termination codon (PTC) (Lachance-Touchette et al., 2011). The premature translation termination is likely to cause mRNA degradation by a process called nonsense-mediated mRNA decay (Baker and Parker, 2004), thereby explaining why expression of α1-K353delins18X did not affect the phenotype of *GABRA1*^−/−^ neurons.

All together, our data suggest that a “loss-of-function” model may not always explain the effects of *GABRA1* mutations on dendritic spines and GABAergic bouton formation. For example, reduced inhibition is most often mentioned as a cause of epileptic syndromes. Here, our data suggest that α1-A322D may instead increase the number of dendritic spines, which are the preferential site of excitatory synapse formation, an event that may result in higher cortical excitation. These potential effects on developing neuronal networks need to be further explored by recording miniature inhibitory (mIPSCs) and excitatory (mEPSCs) postsynaptic currents in transfected neurons.

With the advance in the technology of large-scale multiplex sequencing or next-generation sequencing (NGS), it is now possible to obtain the sequence of the whole exome (WES) and even the whole genome (WGS) for a given individual. These methodological approaches are very powerful and are already opening new frontiers of genomics research. However, by sequencing many more genomes, we will need *in vitro* models to determine the functional biological role of all new variants that we will find. The use of heterelogs models such as HEK cells and xenopus oocytes may not be the best systems to test the impact of mutations in GABA_A_R subunits. For example, despite a large number of studies, the functional alterations caused by missenses mutations identified in *GABRG2* in epileptic patients are still not well understood. In fact, there is no consensus about the effect of the mutations R82Q, P83S, R177G, and K328M on the GABA currents amplitude (Baulac et al., 2001; Wallace et al., 2001; Bianchi et al., 2002; Bowser et al., 2002; Kang and Macdonald, 2004; Hales et al., 2005; Audenaert et al., 2006; Kang et al., 2006; Eugene et al., 2007; Frugier et al., 2007; Goldschen-Ohm et al., 2010; Lachance-Touchette et al., 2011; Huang et al., 2014; Todd et al., 2014). As another example, it is still debated whether β 3-P11S, β 3-G32R, and γ2-P83S altered surface expression of the GABA_A_R or of the subunit itself (Tanaka et al., 2008; Delahanty et al., 2011; Lachance-Touchette et al., 2011; Gurba et al., 2012). In addition, the exclusive use of non-neuronal cells will not answer the question on how biophysical alterations in mutated receptor properties may alter brain development and ultimately lead to hyperexcitable networks. Here, we suggest that organotypic slice cultures may provide an accessible system for investigating the specific effects of GABA receptor mutants on neuronal development. Moreover, in contrast to what occur in dissociated neuronal cultures, organotypic slice cultures retain complex 3-dimensional interactions between different cell types as they occur *in vivo*. Therefore, we believe that the single cell genetic manipulation described here is a novel tool to understand how GABA_A_ receptor mutants disrupt neuronal circuit formation and will help define the pathophysiology of genetic epilepsy syndromes.

### Conflict of interest statement

The authors declare that the research was conducted in the absence of any commercial or financial relationships that could be construed as a potential conflict of interest.

## References

[B1] Arain F. M. Boyd K. L. Gallagher M. J. (2012). Decreased viability and absence-like epilepsy in mice lacking or deficient in the GABAA receptor alpha1 subunit. Epilepsia 53, e161–e165 10.1111/j.1528-1167.2012.03596.x 22812724 PMC3418418

[B2] Audenaert D. Schwartz E. Claeys K. G. Claes L. Deprez L. Suls A. (2006). A novel GABRG2 mutation associated with febrile seizures. Neurology 67, 687–690 10.1212/01.wnl.0000230145.73496.a2 16924025

[B3] Baho E. Di Cristo G. (2012). Neural activity and neurotransmission regulate the maturation of the innervation field of cortical GABAergic interneurons in an age-dependent manner. J. Neurosci. 32, 911–918 10.1523/JNEUROSCI.4352-11.2012 22262889 PMC6621145

[B4] Baker K. E. Parker R. (2004). Nonsense-mediated mRNA decay: terminating erroneous gene expression. Curr. Opin. Cell Biol. 16, 293–299 10.1016/j.ceb.2004.03.003 15145354

[B5] Baulac S. Huberfeld G. Gourfinkel-An I. Mitropoulou G. Beranger A. Prud'homme J. F. (2001). First genetic evidence of GABA(A) receptor dysfunction in epilepsy: a mutation in the gamma2-subunit gene. Nat. Genet. 28, 46–48 10.1038/ng0501-46 11326274

[B6] Bernard C. (2012). Alterations in synaptic function in epilepsy, in Jasper's Basic Mechanisms of the Epilepsies, 4th edn., eds Noebels J. L. Avoli M. Rogawski M. A. Olsen R. W. Delgado-Escueta A. V. (Bethesda, MD: Oxford University Press, Inc.), 470–483

[B7] Bianchi M. T. Song L. Zhang H. Macdonald R. L. (2002). Two different mechanisms of disinhibition produced by GABAA receptor mutations linked to epilepsy in humans. J. Neurosci. 22, 5321–5327 12097483 10.1523/JNEUROSCI.22-13-05321.2002 PMC6758211

[B8] Bosman L. W. Heinen K. Spijker S. Brussaard A. B. (2005). Mice lacking the major adult GABAA receptor subtype have normal number of synapses, but retain juvenile IPSC kinetics until adulthood. J. Neurophysiol. 94, 338–346 10.1152/jn.00084.2005 15758057

[B9] Bowser D. N. Wagner D. A. Czajkowski C. Cromer B. A. Parker M. W. Wallace R. H. (2002). Altered kinetics and benzodiazepine sensitivity of a GABAA receptor subunit mutation [gamma 2(R43Q)] found in human epilepsy. Proc. Natl. Acad. Sci. U.S.A. 99, 15170–15175 10.1073/pnas.212320199 12415111 PMC137562

[B10] Carlson G. Wang Y. Alger B. E. (2002). Endocannabinoids facilitate the induction of LTP in the hippocampus. Nat. Neurosci. 5, 723–724 10.1038/nn879 12080342

[B11] Carvill G. L. Heavin S. B. Yendle S. C. McMahon J. M. O'roak B. J. Cook J. (2013). Targeted resequencing in epileptic encephalopathies identifies *de novo* mutations in CHD2 and SYNGAP1. Nat. Genet. 45, 825–830 10.1038/ng.2646 23708187 PMC3704157

[B12] Carvill G. L. Weckhuysen S. McMahon J. M. Hartmann C. Moller R. S. Hjalgrim H. (2014). *GABRA1* and *STXBP1*: novel genetic causes of Dravet syndrome. Neurology 82, 1245–1253 10.1212/WNL.0000000000000291 24623842 PMC4001207

[B13] Chattopadhyaya B. Baho E. Huang Z. J. Schachner M. Di Cristo G. (2013). Neural cell adhesion molecule-mediated Fyn activation promotes GABAergic synapse maturation in postnatal mouse cortex. J. Neurosci. 33, 5957–5968 10.1523/JNEUROSCI.1306-12.2013 23554477 PMC6618917

[B14] Chattopadhyaya B. Di Cristo G. Higashiyama H. Knott G. W. Kuhlman S. J. Welker E. (2004). Experience and activity-dependent maturation of perisomatic GABAergic innervation in primary visual cortex during a postnatal critical period. J. Neurosci. 24, 9598–9611 10.1523/JNEUROSCI.1851-04.2004 15509747 PMC6730138

[B15] Chattopadhyaya B. Di Cristo G. Wu C. Z. Knott G. Kuhlman S. Fu Y. (2007). GAD67-mediated GABA synthesis and signaling regulate inhibitory synaptic innervation in the visual cortex. Neuron 54, 889–903 10.1016/j.neuron.2007.05.015 17582330 PMC2077924

[B16] Cossette P. Lachance-Touchette P. Rouleau G. A. (2012). Mutated GABAA receptor subunits in idiopathic generalized epilepsy, in Jasper's Basic Mechanisms of the Epilepsies, 4th edn., eds Noebels J. L. Avoli M. Rogawski M. A. Olsen R. W. Delgado-Escueta A. V. (Bethesda, MD: Oxford University Press, Inc.), 714–730 22787675

[B17] Cossette P. Liu L. Brisebois K. Dong H. Lortie A. Vanasse M. (2002). Mutation of *GABRA1* in an autosomal dominant form of juvenile myoclonic epilepsy. Nat. Genet. 31, 184–189 10.1038/ng885 11992121

[B18] Delahanty R. J. Kang J. Q. Brune C. W. Kistner E. O. Courchesne E. Cox N. J. (2011). Maternal transmission of a rare GABRB3 signal peptide variant is associated with autism. Mol. Psychiatry 16, 86–96 10.1038/mp.2009.118 19935738 PMC3428055

[B19] Dibbens L. M. Feng H. J. Richards M. C. Harkin L. A. Hodgson B. L. Scott D. (2004). GABRD encoding a protein for extra- or peri-synaptic GABAA receptors is a susceptibility locus for generalized epilepsies. Hum. Mol. Genet. 13, 1315–1319 10.1093/hmg/ddh146 15115768

[B20] Dibbens L. M. Harkin L. A. Richards M. Hodgson B. L. Clarke A. L. Petrou S. (2009). The role of neuronal GABA(A) receptor subunit mutations in idiopathic generalized epilepsies. Neurosci. Lett. 453, 162–165 10.1016/j.neulet.2009.02.038 19429026

[B21] Di Cristo G. Chattopadhyaya B. Kuhlman S. J. Fu Y. Belanger M. C. Wu C. Z. (2007). Activity-dependent PSA expression regulates inhibitory maturation and onset of critical period plasticity. Nat. Neurosci. 10, 1569–1577 10.1038/nn2008 18026099

[B22] Di Cristo G. Wu C. Chattopadhyaya B. Ango F. Knott G. Welker E. (2004). Subcellular domain-restricted GABAergic innervation in primary visual cortex in the absence of sensory and thalamic inputs. Nat. Neurosci. 7, 1184–1186 10.1038/nn1334 15475951

[B23] Ding L. Feng H. J. Macdonald R. L. Botzolakis E. J. Hu N. Gallagher M. J. (2010). GABA(A) receptor alpha1 subunit mutation A322D associated with autosomal dominant juvenile myoclonic epilepsy reduces the expression and alters the composition of wild type GABA(A) receptors. J. Biol. Chem. 285, 26390–26405 10.1074/jbc.M110.142299 20551311 PMC2924069

[B24] Dunaevsky A. Tashiro A. Majewska A. Mason C. Yuste R. (1999). Developmental regulation of spine motility in the mammalian central nervous system. Proc. Natl. Acad. Sci. U.S.A. 96, 13438–13443 10.1073/pnas.96.23.13438 10557339 PMC23966

[B25] Epi K. C. Epilepsy Phenome/Genome P. Allen A. S. Berkovic S. F. Cossette P. Delanty N. (2013). *De novo* mutations in epileptic encephalopathies. Nature 501, 217–221 10.1038/nature12439 23934111 PMC3773011

[B26] Eugene E. Depienne C. Baulac S. Baulac M. Fritschy J. M. Le Guern E. (2007). GABA(A) receptor gamma 2 subunit mutations linked to human epileptic syndromes differentially affect phasic and tonic inhibition. J. Neurosci. 27, 14108–14116 10.1523/JNEUROSCI.2618-07.2007 18094250 PMC6673514

[B27] Fritschy J. M. Panzanelli P. (2006). Molecular and synaptic organization of GABAA receptors in the cerebellum: effects of targeted subunit gene deletions. Cerebellum 5, 275–285 10.1080/14734220600962805 17134990

[B28] Frugier G. Coussen F. Giraud M. F. Odessa M. F. Emerit M. B. Boue-Grabot E. (2007). A gamma 2(R43Q) mutation, linked to epilepsy in humans, alters GABAA receptor assembly and modifies subunit composition on the cell surface. J. Biol. Chem. 282, 3819–3828 10.1074/jbc.M608910200 17148443

[B29] Gaiarsa J. L. Porcher C. (2013). Emerging neurotrophic role of GABAB receptors in neuronal circuit development. Front. Cell. Neurosci. 7:206 10.3389/fncel.2013.00206 24282395 PMC3824957

[B30] Gallagher M. J. Ding L. Maheshwari A. Macdonald R. L. (2007). The GABAA receptor alpha1 subunit epilepsy mutation A322D inhibits transmembrane helix formation and causes proteasomal degradation. Proc. Natl. Acad. Sci. U.S.A. 104, 12999–13004 10.1073/pnas.0700163104 17670950 PMC1941799

[B31] Gallagher M. J. Song L. Arain F. Macdonald R. L. (2004). The juvenile myoclonic epilepsy GABA(A) receptor alpha1 subunit mutation A322D produces asymmetrical, subunit position-dependent reduction of heterozygous receptor currents and alpha1 subunit protein expression. J. Neurosci. 24, 5570–5578 10.1523/JNEUROSCI.1301-04.2004 15201329 PMC6729321

[B32] Goldschen-Ohm M. P. Wagner D. A. Petrou S. Jones M. V. (2010). An epilepsy-related region in the GABA(A) receptor mediates long-distance effects on GABA and benzodiazepine binding sites. Mol. Pharmacol. 77, 35–45 10.1124/mol.109.058289 19846749 PMC2802431

[B33] Gurba K. N. Hernandez C. C. Hu N. Macdonald R. L. (2012). GABRB3 mutation, G32R, associated with childhood absence epilepsy alters alpha1beta3gamma2L gamma-aminobutyric acid type A (GABAA) receptor expression and channel gating. J. Biol. Chem. 287, 12083–12097 10.1074/jbc.M111.332528 22303015 PMC3320954

[B34] Haider B. McCormick D. A. (2009). Rapid neocortical dynamics: cellular and network mechanisms. Neuron 62, 171–189 10.1016/j.neuron.2009.04.008 19409263 PMC3132648

[B35] Hales T. G. Tang H. Bollan K. A. Johnson S. J. King D. P. McDonald N. A. (2005). The epilepsy mutation, gamma2(R43Q) disrupts a highly conserved inter-subunit contact site, perturbing the biogenesis of GABAA receptors. Mol. Cell. Neurosci. 29, 120–127 10.1016/j.mcn.2005.01.002 15866052

[B36] Hancili S. Onal Z. E. Ata P. Karatoprak E. Y. Gurbuz T. Bostanci M. (2014). The GABAA receptor gamma2 subunit (R43Q) mutation in febrile seizures. Pediatr. Neurol. 50, 353–356 10.1016/j.pediatrneurol.2014.01.002 24630281

[B37] Harkin L. A. Bowser D. N. Dibbens L. M. Singh R. Phillips F. Wallace R. H. (2002). Truncation of the GABA(A)-receptor gamma2 subunit in a family with generalized epilepsy with febrile seizures plus. Am. J. Hum. Genet. 70, 530–536 10.1086/338710 11748509 PMC384926

[B77] Hayama T. Noguchi J. Watanabe S. Takahashi N. Hayashi-Takagi A. Ellis-Davies G. C. (2013). GABA promotes the competitive selection of dendritic spines by controlling local Ca2+ signaling. Nat. Neurosci. 16, 1409–1416 10.1038/nn.3496 23974706 PMC4135703

[B38] Huang X. Hernandez C. C. Hu N. Macdonald R. L. (2014). Three epilepsy-associated GABRG2 missense mutations at the gamma+/beta- interface disrupt GABA receptor assembly and trafficking by similar mechanisms but to different extents. Neurobiol. Dis. 68C, 167–179 10.1016/j.nbd.2014.04.015 24798517 PMC4169075

[B39] Ishii A. Kanaumi T. Sohda M. Misumi Y. Zhang B. Kakinuma N. (2014). Association of nonsense mutation in GABRG2 with abnormal trafficking of GABAA receptors in severe epilepsy. Epilepsy Res. 108, 420–432 10.1016/j.eplepsyres.2013.12.005 24480790

[B40] Isokawa M. (2000). Remodeling dendritic spines of dentate granule cells in temporal lobe epilepsy patients and the rat pilocarpine model. Epilepsia 41 Suppl. 6, S14–S17 10.1111/j.1528-1157.2000.tb01550.x 10999513

[B41] Johnston A. J. Kang J. Q. Shen W. Pickrell W. O. Cushion T. D. Davies J. S. (2014). A novel GABRG2 mutation, p.R136^*^, in a family with GEFS+ and extended phenotypes. Neurobiol. Dis. 64, 131–141 10.1016/j.nbd.2013.12.013 24407264 PMC4222744

[B42] Kananura C. Haug K. Sander T. Runge U. Gu W. Hallmann K. (2002). A splice-site mutation in GABRG2 associated with childhood absence epilepsy and febrile convulsions. Arch. Neurol. 59, 1137–1141 10.1001/archneur.59.7.1137 12117362

[B43] Kang J. Q. Macdonald R. L. (2004). The GABAA receptor gamma2 subunit R43Q mutation linked to childhood absence epilepsy and febrile seizures causes retention of alpha1beta2gamma2S receptors in the endoplasmic reticulum. J. Neurosci. 24, 8672–8677 10.1523/JNEUROSCI.2717-04.2004 15470132 PMC6729953

[B44] Kang J. Q. Shen W. Macdonald R. L. (2006). Why does fever trigger febrile seizures? GABAA receptor gamma2 subunit mutations associated with idiopathic generalized epilepsies have temperature-dependent trafficking deficiencies. J. Neurosci. 26, 2590–2597 10.1523/JNEUROSCI.4243-05.2006 16510738 PMC6793669

[B45] Kilb W. Kirischuk S. Luhmann H. J. (2013). Role of tonic GABAergic currents during pre- and early postnatal rodent development. Front. Neural Circuits 7:139 10.3389/fncir.2013.00139 24027498 PMC3760143

[B46] Klassen T. Davis C. Goldman A. Burgess D. Chen T. Wheeler D. (2011). Exome sequencing of ion channel genes reveals complex profiles confounding personal risk assessment in epilepsy. Cell 145, 1036–1048 10.1016/j.cell.2011.05.025 21703448 PMC3131217

[B47] Kralic J. E. Sidler C. Parpan F. Homanics G. E. Morrow A. L. Fritschy J. M. (2006). Compensatory alteration of inhibitory synaptic circuits in cerebellum and thalamus of gamma-aminobutyric acid type A receptor alpha1 subunit knockout mice. J. Comp. Neurol. 495, 408–421 10.1002/cne.20866 16485284

[B48] Krampfl K. Maljevic S. Cossette P. Ziegler E. Rouleau G. A. Lerche H. (2005). Molecular analysis of the A322D mutation in the GABA receptor alpha-subunit causing juvenile myoclonic epilepsy. Eur. J. Neurosci. 22, 10–20 10.1111/j.1460-9568.2005.04168.x 16029191

[B49] Lachance-Touchette P. Brown P. Meloche C. Kinirons P. Lapointe L. Lacasse H. (2011). Novel alpha1 and gamma2 GABAA receptor subunit mutations in families with idiopathic generalized epilepsy. Eur. J. Neurosci. 34, 237–249 10.1111/j.1460-9568.2011.07767.x 21714819

[B50] Lachance-Touchette P. Martin C. Poulin C. Gravel M. Carmant L. Cossette P. (2010). Screening of GABRB3 in French-Canadian families with idiopathic generalized epilepsy. Epilepsia 51, 1894–1897 10.1111/j.1528-1167.2010.02642.x 20550555

[B51] Lee K. F. Soares C. Beique J. C. (2012). Examining form and function of dendritic spines. Neural Plast. 2012:704103 10.1155/2012/704103 22577585 PMC3345238

[B52] Li M. Z. Elledge S. J. (2007). Harnessing homologous recombination *in vitro* to generate recombinant DNA via SLIC. Nat. Methods 4, 251–256 10.1038/nmeth1010 17293868

[B53] Liu Q. S. Pu L. Poo M. M. (2005). Repeated cocaine exposure *in vivo* facilitates LTP induction in midbrain dopamine neurons. Nature 437, 1027–1031 10.1038/nature04050 16222299 PMC1457101

[B54] Luscher C. Nicoll R. A. Malenka R. C. Muller D. (2000). Synaptic plasticity and dynamic modulation of the postsynaptic membrane. Nat. Neurosci. 3, 545–550 10.1038/75714 10816309

[B55] Ma Y. Ramachandran A. Ford N. Parada I. Prince D. A. (2013). Remodeling of dendrites and spines in the C1q knockout model of genetic epilepsy. Epilepsia 54, 1232–1239 10.1111/epi.12195 23621154 PMC3700654

[B56] Macdonald R. L. Kang J. Q. (2009). Molecular pathology of genetic epilepsies associated with GABAA receptor subunit mutations. Epilepsy Curr. 9, 18–23 10.1111/j.1535-7511.2008.01278.x 19396344 PMC2668111

[B57] Macdonald R. L. Kang J. Q. Gallagher M. J. (2010). Mutations in GABAA receptor subunits associated with genetic epilepsies. J. Physiol. 588, 1861–1869 10.1113/jphysiol.2010.186999 20308251 PMC2901974

[B58] Maljevic S. Krampfl K. Cobilanschi J. Tilgen N. Beyer S. Weber Y. G. (2006). A mutation in the GABA(A) receptor alpha(1)-subunit is associated with absence epilepsy. Ann. Neurol. 59, 983–987 10.1002/ana.20874 16718694

[B59] Multani P. Myers R. H. Blume H. W. Schomer D. L. Sotrel A. (1994). Neocortical dendritic pathology in human partial epilepsy: a quantitative Golgi study. Epilepsia 35, 728–736 10.1111/j.1528-1157.1994.tb02503.x 7521835

[B60] Queenan B. N. Lee K. J. Pak D. T. (2012). Wherefore art thou, homeo(stasis)? Functional diversity in homeostatic synaptic plasticity. Neural Plast 2012:718203 10.1155/2012/718203 22685679 PMC3362963

[B61] Rossignol E. (2011). Genetics and function of neocortical GABAergic interneurons in neurodevelopmental disorders. Neural Plast. 2011:649325 10.1155/2011/649325 21876820 PMC3159129

[B62] Shi X. Huang M. C. Ishii A. Yoshida S. Okada M. Morita K. (2010). Mutational analysis of GABRG2 in a Japanese cohort with childhood epilepsies. J. Hum. Genet. 55, 375–378 10.1038/jhg.2010.47 20485450

[B63] Sieghart W. Sperk G. (2002). Subunit composition, distribution and function of GABA(A) receptor subtypes. Curr. Top. Med. Chem. 2, 795–816 10.2174/1568026023393507 12171572

[B78] Stoppini L. Buchs P. A. Muller D. (1991). A simple method for organotypic cultures of nervous tissue. J. Neurosci. Methods 37, 173–182 10.1016/0165-0270(91)90128-M 1715499

[B64] Sun H. Zhang Y. Liang J. Liu X. Ma X. Wu H. (2008). SCN1A, SCN1B, and GABRG2 gene mutation analysis in Chinese families with generalized epilepsy with febrile seizures plus. J. Hum. Genet. 53, 769–774 10.1007/s10038-008-0306-y 18566737

[B65] Tan H. O. Reid C. A. Single F. N. Davies P. J. Chiu C. Murphy S. (2007). Reduced cortical inhibition in a mouse model of familial childhood absence epilepsy. Proc. Natl. Acad. Sci. U.S.A. 104, 17536–17541 10.1073/pnas.0708440104 17947380 PMC2077291

[B66] Tanaka M. Olsen R. W. Medina M. T. Schwartz E. Alonso M. E. Duron R. M. (2008). Hyperglycosylation and reduced GABA currents of mutated GABRB3 polypeptide in remitting childhood absence epilepsy. Am. J. Hum. Genet. 82, 1249–1261 10.1016/j.ajhg.2008.04.020 18514161 PMC2427288

[B67] Tian M. Mei D. Freri E. Hernandez C. C. Granata T. Shen W. (2013). Impaired surface alphabetagamma GABA(A) receptor expression in familial epilepsy due to a GABRG2 frameshift mutation. Neurobiol. Dis. 50, 135–141 10.1016/j.nbd.2012.10.008 23069679 PMC3762699

[B68] Todd E. Gurba K. N. Botzolakis E. J. Stanic A. K. Macdonald R. L. (2014). GABAA receptor biogenesis is impaired by the gamma2 subunit febrile seizure-associated mutation, GABRG2(R177G). Neurobiol. Dis. 69, 215–224 10.1016/j.nbd.2014.05.013 24874541

[B69] Trevelyan A. J. Baldeweg T. Van Drongelen W. Yuste R. Whittington M. (2007). The source of afterdischarge activity in neocortical tonic-clonic epilepsy. J. Neurosci. 27, 13513–13519 10.1523/JNEUROSCI.3005-07.2007 18057209 PMC6673106

[B70] Vicini S. Ferguson C. Prybylowski K. Kralic J. Morrow A. L. Homanics G. E. (2001). GABA(A) receptor alpha1 subunit deletion prevents developmental changes of inhibitory synaptic currents in cerebellar neurons. J. Neurosci. 21, 3009–3016 11312285 10.1523/JNEUROSCI.21-09-03009.2001 PMC6762566

[B71] Wallace R. H. Marini C. Petrou S. Harkin L. A. Bowser D. N. Panchal R. G. (2001). Mutant GABA(A) receptor gamma2-subunit in childhood absence epilepsy and febrile seizures. Nat. Genet. 28, 49–52 10.1038/ng0501-49 11326275

[B72] Whissell P. D. Eng D. Lecker I. Martin L. J. Wang D. S. Orser B. A. (2013). Acutely increasing deltaGABA(A) receptor activity impairs memory and inhibits synaptic plasticity in the hippocampus. Front. Neural Circuits 7:146 10.3389/fncir.2013.00146 24062648 PMC3775149

[B73] Wong M. (2005). Modulation of dendritic spines in epilepsy: cellular mechanisms and functional implications. Epilepsy Behav. 7, 569–577 10.1016/j.yebeh.2005.08.007 16246628

[B74] Wu X. Fu Y. Knott G. Lu J. Di Cristo G. Huang Z. J. (2012). GABA signaling promotes synapse elimination and axon pruning in developing cortical inhibitory interneurons. J. Neurosci. 32, 331–343 10.1523/JNEUROSCI.3189-11.2012 22219294 PMC3742883

[B75] Zeller A. Jurd R. Lambert S. Arras M. Drexler B. Grashoff C. (2008). Inhibitory ligand-gated ion channels as substrates for general anesthetic actions. Handb. Exp. Pharmacol. 182, 31–51 10.1007/978-3-540-74806-9_2 18175085

