# Supplementary material for: Single-cell genetic expression of mutant GABAA receptors causing Human genetic epilepsy alters dendritic spine and GABAergic bouton formation in a mutation-specific manner
Source: Front Cell Neurosci. 2014 Oct 14;8:317. doi: 10.3389/fncel.2014.00317 (PMC4196543; doi:10.3389/fncel.2014.00317)
Supplement: Supplementary file 1 [file DataSheet1.DOCX]

***Supplementary Material***

**Single-cell genetic expression of mutant GABA_A_ receptors causing Human genetic epilepsy alters dendritic spine and GABAergic bouton formation in a mutation-specific manner**

**A Pamela Lachance-Touchette^1*^, Mayukh Choudhury^2*^, Ana Stoica^1^, Graziella Di Cristo^2$^ & Patrick Cossette^1$^**

^1^Centre d’Excellence en Neuromique de l’Université de Montréal (CENUM), Centre de recherche CHUM, Montréal, QC, Canada

^2^Centre Hospitalier Universitaire Sainte-Justine, Université de Montréal, Montréal, QC, Canada

*Both authors contributed equally to this study

**Correspondence:**

$-Graziella Di Cristo, CHU Ste-Justine, 3175, Chemin de la Côte-Ste-Catherine, Montréal (Quebec), H3T 1C5, E-mail: [graziella.di.cristo@recherche-ste-justine.qc.ca](mailto:graziella.di.cristo@recherche-ste-justine.qc.ca).

$-Dr. Patrick Cossette, CHUM-Hôpital Notre-Dame, 1560 Sherbrooke est, Montréal, Québec, H2L 4M1, E-mail: [patrick.cossette@umontreal.ca](mailto:patrick.cossette@umontreal.ca).

1. **Supplementary Figures and Tables**

## Suplementary Figures


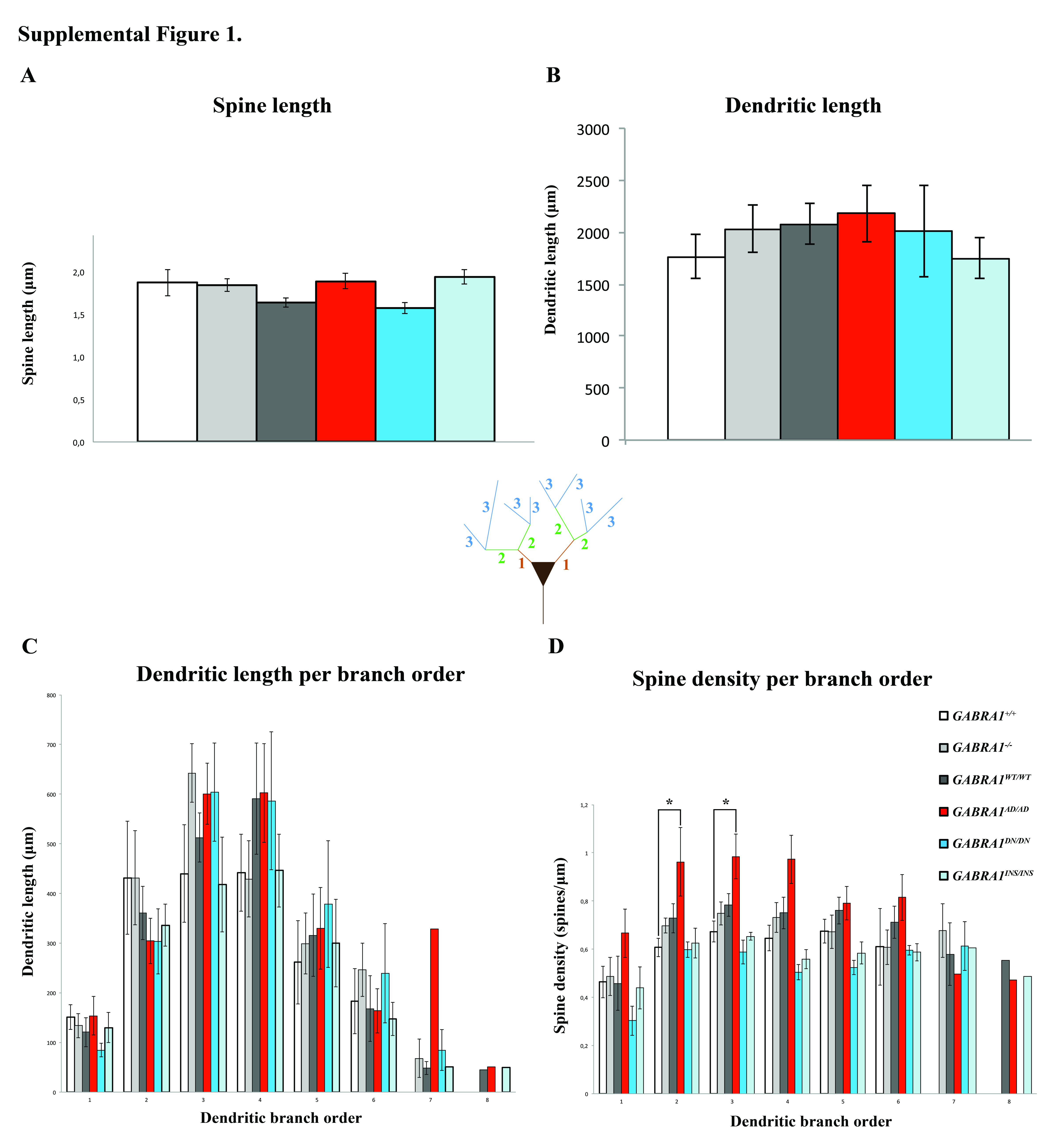


**Supplementary Figure 1. α1-A322D expression induces a significant increase in spines density in cortical pyramidal cells.** (**A**) Spine length, (**B**) dendritic length and (**C**) dendritic length per branch order do not differ across the groups. (**D**) *GABRA1^AD/AD^* mutant pyramidal cells show significantly increased density of spines in dendritic branch order two and three compared to control age-matched pyramidal cells *GABRA^+/+^* (1-way ANOVA; *p<0.05). GFP n=7; GFP-CRE n=9; GFP-CRE-WT n=7; GFP-CRE-A322D n=7; GFP-CRE-D219N n=6; GFP-CRE-K353delins18X n=7.

**
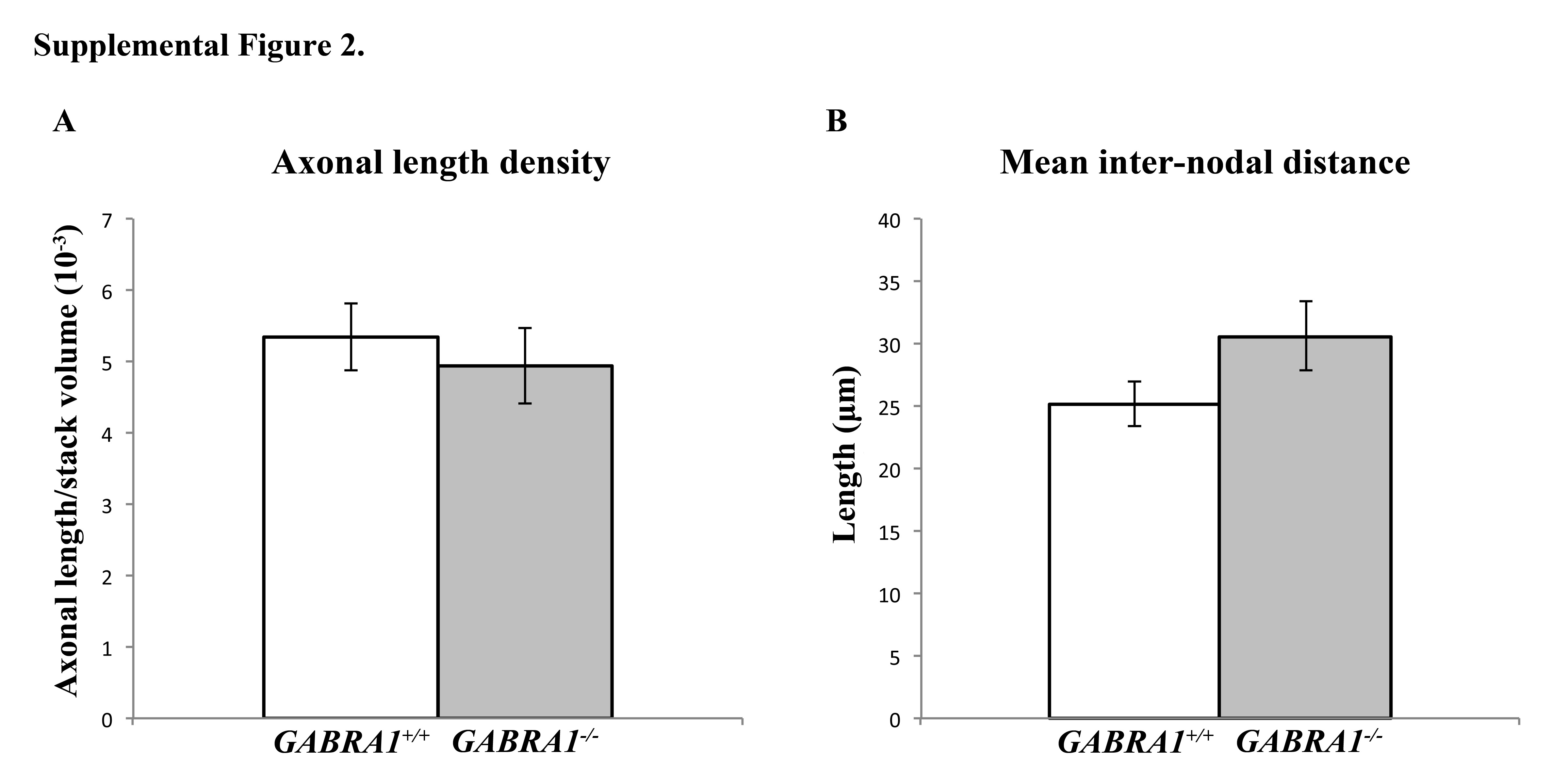
**

**Supplementary Figure 2. *GABRA1* knockdown does not alter the overall axon growth basket cells.** (A) Axonal density and (B) inter-node axon length are not significantly different between *GABRA1^-/-^* and *GABRA1^+/+^* basket cells. GFP n=6; GFP-CRE, n=6 basket cells.
